# Lifestyle Activities and Incident Dementia from Claims in the Baltimore Experience Corps Study

**DOI:** 10.1093/geroni/igaf122.3803

**Published:** 2025-12-31

**Authors:** Kyle Moored, Vishaldeep Sekhon, George Rebok, Kate Gordon, Jeanine Parisi, Qian-Li Xue, Michelle Carlson

**Affiliations:** Johns Hopkins Bloomberg School of Public Health, Baltimore, Maryland, United States; Johns Hopkins University School of Medicine, Baltimore, Maryland, United States; Johns Hopkins University, Baltimore, Maryland, United States; Johns Hopkins Bloomberg School of Public Health, Baltimore, Maryland, United States; Johns Hopkins Bloomberg School of Public Health, Baltimore, Maryland, United States; Johns Hopkins University, Baltimore, Maryland, United States; Johns Hopkins Bloomberg School of Public Health, Baltimore, Maryland, United States

## Abstract

Lifestyle activity engagement (e.g., social clubs, classes) in later life has been linked with reduced risk of dementia, but studies have often been limited to short follow-up periods. We examined whether variety and frequency of activity engagement predicted incident dementia risk, leveraging 15 years of linked longitudinal claims data in the Baltimore Experience Corps Study (BECS). Participants were 520 individuals from BECS (2006-2012) with available claims data (mean age=68.2, 84% women, 95% Black/African American). Participants self-reported baseline engagement in 27 activities, and their total number of activities (activity variety) and average frequency of engagement across activities (activity frequency) were calculated. Incident dementia diagnoses (2008-2022) were derived from linked CMS and private claims using the Bynum algorithm. We used Cox proportional hazards models, adjusting for age, sex, education, and intervention group. Participants reported an average baseline variety of 18.8 activities (SD = 3.3) and average activity frequency of 8.8 days per month (SD = 2.6). Hazard rates of incident dementia were 5.2% lower for each additional activity (activity variety: HR=.948, 95% CI: 0.89-1.00), which was slightly attenuated after further adjusting for baseline health conditions and depression (HR=.953, 95% CI: 0.90-1.01). Results were similar in magnitude but not statistically significant for activity frequency (p>.05). In a sample of primarily Black older adults, higher activity variety in later life predicted lower dementia risk over 15 years. Importantly, lifestyle activity engagement was previously shown to be increased by the Experience Corps intervention, suggesting that it may be a potentially modifiable target to promote cognitive resilience with aging.

